# Craniopharyngioma involving the anterior, middle, and posterior cranial fossa in adults: A case report

**DOI:** 10.3389/fneur.2023.1098600

**Published:** 2023-01-26

**Authors:** Bin Tian, Ming Li, Xiaolin Du, Hui Zhou, Kun Zhou, Shiguang Li

**Affiliations:** ^1^Department of Radiology, The Second People's Hospital of Guiyang (Jinyang Hospital), Guiyang, Guizhou, China; ^2^Department of Pathology, The Second People's Hospital of Guiyang (Jinyang Hospital), Guiyang, Guizhou, China; ^3^Department of Neurosurgery, The Second People's Hospital of Guiyang (Jinyang Hospital), Guiyang, Guizhou, China

**Keywords:** craniopharyngioma, multiple cystic masses, adult, CT, MRI

## Abstract

Craniopharyngioma (CP) is a rare benign tumor that develops from the residual epithelial cells of the craniopharynx, accounting for < 5% of intracranial tumors. It is common for CPs to grow in the sellar/parasellar region and extend suprasellar. The pathology classifies CPs into adamantinomatous craniopharyngiomas (ACP) and papillary craniopharyngiomas (PCP). The PCP is mainly solid and occurs only in adults. ACP is predominantly cystic and more common in childhood and adolescent. Multilocular cystic ACP involving the anterior, middle, and posterior cranial fossa is rare in adults. Here, we report a case of a 46-year-old adult male patient who presented with recurrent headaches for 1 year with choking and hoarseness. Computed tomography (CT) and magnetic resonance imaging (MRI) revealed multiple cystic masses in the anterior, middle, and posterior cranial fossa. Initial hypotheses included the following: CP, colloid cyst, enterogenous cyst, epidermoid cyst, and dermoid cyst. Subsequently, the patient underwent surgery and postoperative histopathology diagnosed ACP. Adults with ACP involving the anterior, middle, and posterior cranial fossae are uncommon. This is a rare condition that radiologists should be aware of.

## Case presentation

**Figure 1:**
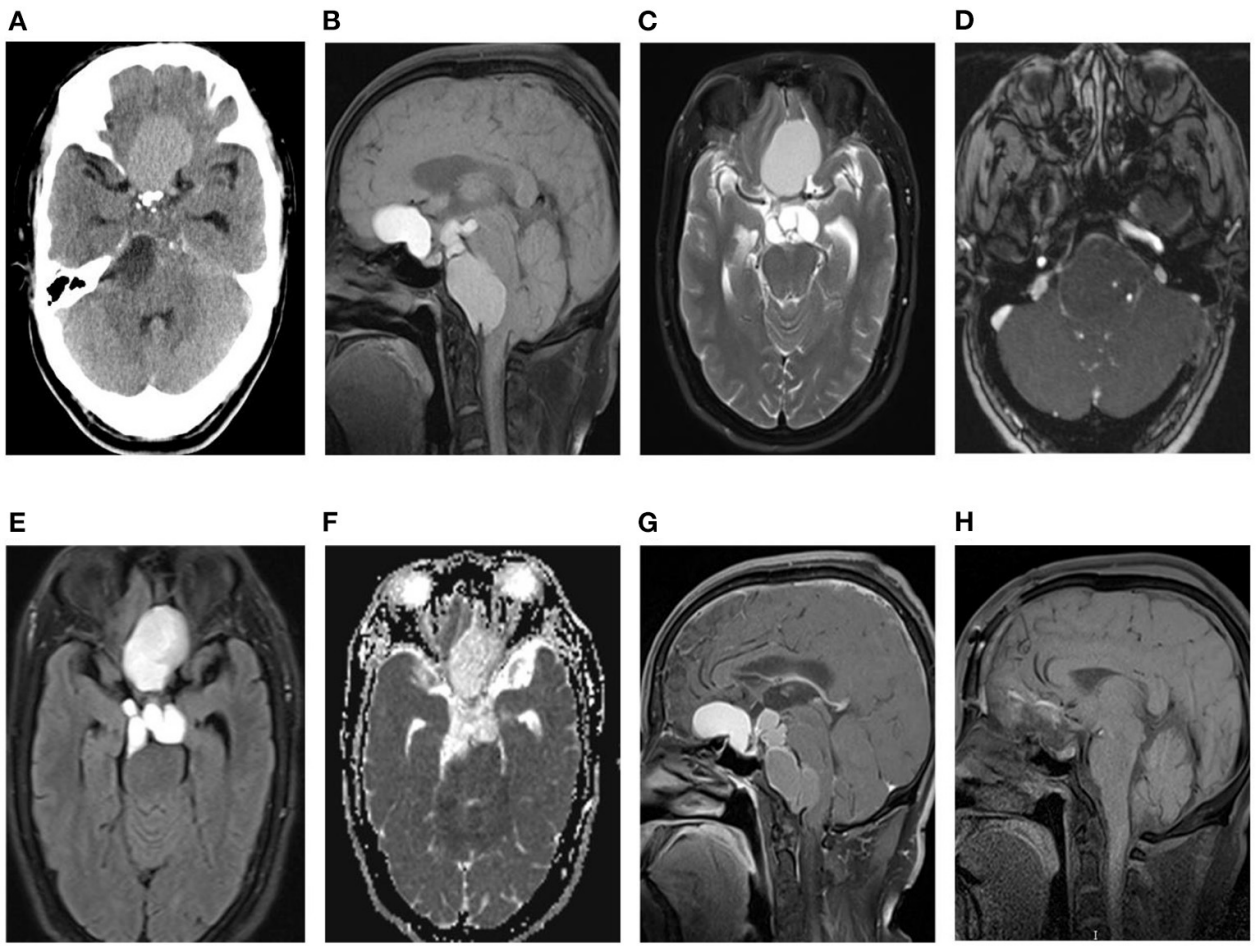
**(A)** Axial CT without contrast in the cerebral window reveals craniopharyngiomas in the anterior, medial, and posterior fossa, with slightly high density in the anterior fossa, slightly low density in middle fossa, low density in the posterior fossa, and small high-density calcifications at the margins of middle fossa tumors. **(B, C)** The anterior, middle, and posterior cranial fossa tumors show multilocular cysts, the anterior cranial fossa tumor shows hyperintense on T1WI **(B)** and T2WI **(C)**, and the middle and posterior cranial fossa tumors show equal signal on T1WI and T2WI. **(D)** MRA shows that the tumor in the posterior fossa surrounds the right vertebral artery. **(E)** FLAIR show mixed hyperintense in the anterior cranial fossa mass and hyperintense in the middle and posterior cranial fossa masses. **(F)** ADC does not show a restricted diffusion hypointense. **(G)** The sagittal T1WI shows no enhancement of the anterior cranial fossa tumor, wall enhancement of the middle and posterior cranial fossa tumors, and no enhancement in the center of all tumors. **(H)** Postoperative sagittal MRI showed that the tumor had been removed.

**Figure 2:**
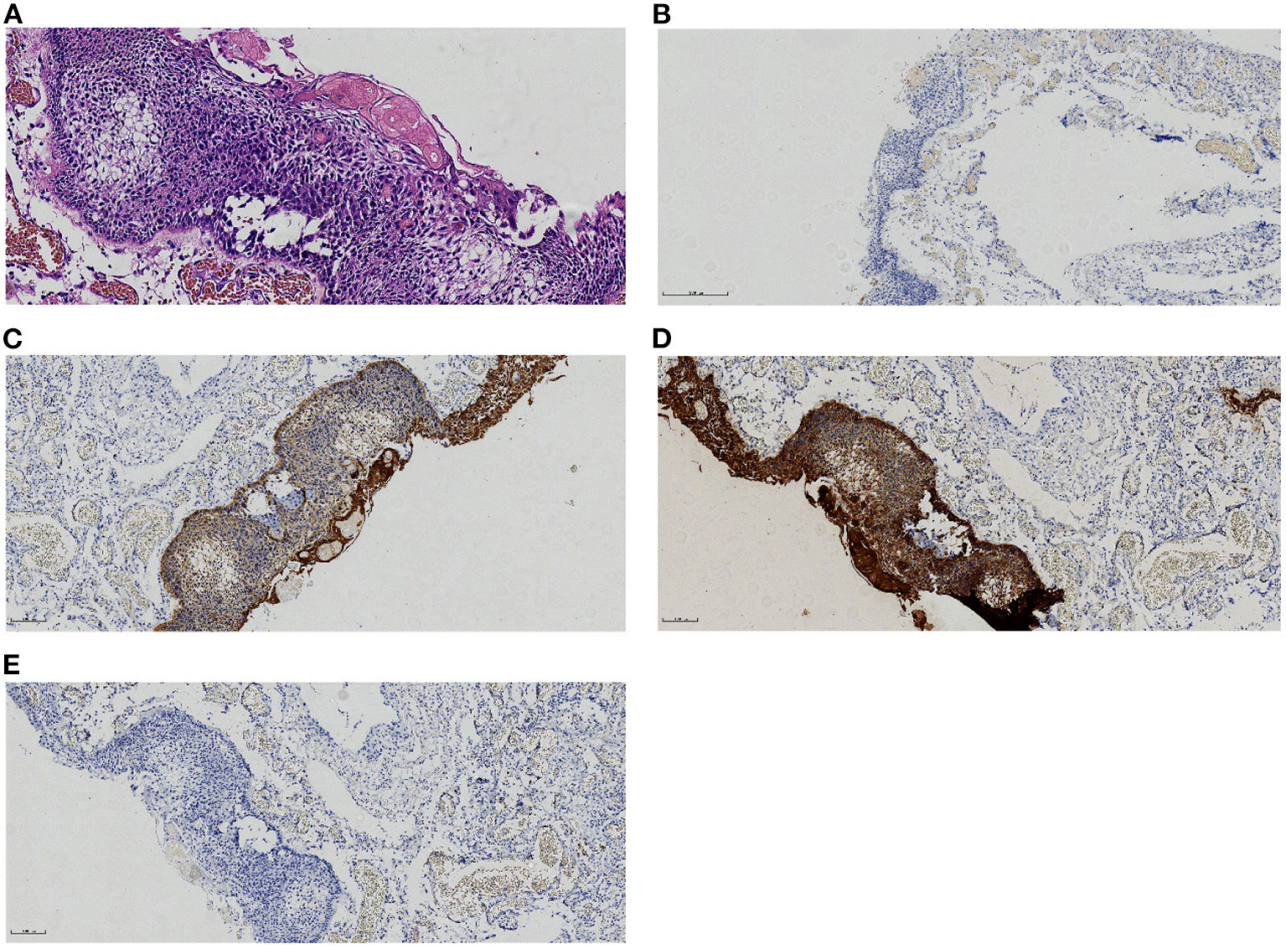
Histological and immunohistochemical features of craniopharyngioma.**(A)** A microscope shows adamantinomatous cells and keratins. **(B–E)** Immunohistochemical staining for CEA-,CK+,CK5/6+,GFAP- [Original magnification: **(A)** 200×;**(B–E)**100×].

A 46-year-old male patient presented with recurrent headaches for 1 year, choking cough, hoarseness of voice without nausea, vomiting, orofacial distortion, and visual field defects. Laboratory test results were normal. CT images of the head suggested multifocal cystic masses in the anterior, middle, and posterior cranial fossa. The tumor was slightly high density in the anterior cranial fossa, slightly high density in the middle cranial fossa with marginal discontinuous calcification, and low density in the posterior cranial fossa (Figure 1A), and there was no involvement of the bilateral cavernous sinuses. MRI images showed that the anterior cranial fossa mass was hyperintense on T1-weighted images (T1WI) and slightly hyperintense on T2-weighted images (T2WI), and the middle and posterior cranial fossa mass was hyperintense on both T1WI and T2WI images (Figures 1B, C). Magnetic resonance angiography (MRA) showed that the posterior cranial fossa mass wrapped around the right vertebral artery (Figure 1D). Fluid-attenuated inversion recovery (FLAIR) images showed that the anterior, middle, and posterior cranial fossa masses were all hyperintense (Figure 1E). There was no restricted diffusion low signal on ADC images (Figure 1F). The contrast-enhanced T1WI images showed no enhancement of the anterior cranial fossa mass wall and significant enhancement of the middle and posterior cranial fossa mass walls, with no enhancement in the center of all masses (Figure 1G). Postoperative MRI images showed that all tumors were removed (Figure 1H). Based on the patient's age, clinical presentation, and imaging features. The radiologists initially proposed five hypotheses: CP, colloid cyst, enterogenous cyst, dermoid cyst, and epidermoid cyst. Due to the large and dispersed nature of the tumor, the anterior, middle, and posterior fossa resection would be more traumatic at the same time, so the neurosurgeon decided to remove the tumor two times. On February 10, 2022, the neurosurgeon performed a mass resection of the middle and posterior fossa via the right retrosigmoid sinus approach. On July 25, 2022, neurosurgeons removed the anterior cranial fossa tumor via a subfrontal interhemispheric approach. The result of the histopathological examination was ACP. Immunohistochemical staining was negative for CEA and GFAP but positive for CK and CK5/6 (Figure 2).

## Discussion

Clinical manifestations of CP include decreased vision, growth retardation, irregular menstruation, increased intracranial pressure, visual field defect, diabetes insipidus, abnormal hormone secretion, and so on. The symptoms of headaches are not specific. When symptoms such as choking and coughing on drinking water, asphyxia, and hoarseness occur, the possible injury of the recurrent laryngeal and superior laryngeal nerve should be considered first. In addition, when the tumor compresses the posterior cranial nerves, it may lead to choking and hoarseness, such as meningioma in the rear cranial fossa. Our patient presented with recurrent headaches for 1 year, choking, coughing on drinking water, and hoarseness.

According to the theory of embryogenesis, CP may occur anywhere in the migration of Rathke's sac (1), such as the nasopharynx, paranasal sinus, third ventricle, and posterior cranial fossa (2, 3). Still, it can also occur in the suprasellar cistern (4), temporal lobe (5), and cavernous sinus (6). This kind of ACP involving the anterior, middle, and posterior cranial fossa of adults is rarely reported in the literature.

World Health Organization (WHO) classified CP into ACP and PCP (7). The PCP mainly occurs in adults; the ACP has a bimodal age distribution, with the peak incidence in children aged 5–15 years and adults aged 45–60 years (8, 9). On CT, CP is predominantly cystic or cystic solid, and solid CP is more common in PCP.ACP is mainly cystic components; the cyst wall with eggshell-like calcification is ACP's characteristic (10–15). On MRI, the cystic part of CP shows a variety of signals due to the different compositions of the cystic fluid. It was usually a hyperintense or slightly hyperintense in T1WI and hyperintense in T2WI. The solid component of CP shows an equal signal on T1WI and equal or slightly hyperintense on T2WI. On diffusion-weighted images (DWI), the solid portion of CP diffused without restricted diffusion, suggesting that CP cells were located in loose connective tissue. MRI with contrast shows marginal enhancement in the cystic part and noticeable enhancement in the solid piece. Still, there is no enhancement in cholesterol crystallization, mineral deposition, and small calcification in the tumor, so there could be fine dots without enhancement in the tumor, which is a grid-like appearance (3, 14, 16). In general, although CP is a benign tumor, it can also undergo malignant changes (17–20). It will also compress and adhesion adjacent nerves and blood vessels to produce symptoms (21–23).

CT and MRI scans are essential to diagnosing and differentially diagnosing CPs. In our case, the density and signal of the anterior, middle, and posterior fossa tumors were diverse, possibly due to differences in protein or crystalline content concentrations in the tumors. MRI contrast-enhanced scan showed a noticeable enhancement of the mass wall of the middle and posterior cranial fossa, indicating that the marginal vessels of the mass in the middle and posterior cranial fossa were abundant. In addition, on CT, small calcification foci were seen along the edge of the tumor wall in the middle cranial fossa. Therefore, the radiologist first hypothesized that it was a craniopharyngioma. However, some of the manifestations in our case are similar to colloid cysts. Colloid cysts showed high density on CT images and hyperintense or hypointense on T2WI but mainly showed hypointense on T2WI and often occurred in the third ventricle (24). The T2WI of this case showed hyperintense, which was not consistent with the typical colloid cyst.

Differentiating CP from intracranial enterogenous cysts, epidermoid cysts, or dermoid cysts is essential. Intracranial enterogenous cysts are very rare, often in the ventral side of the spinal cord, occasionally in the brain parenchyma and clivus of the posterior cranial fossa, and rarely in the suprasellar, quadrigeminal cistern and anterior cranial fossa (25). The T1WI signal of typical enterogenous cysts was equal to or slightly higher than that of cerebrospinal fluid. The signal of T2WI and FLAIR was higher than that of cerebrospinal fluid. There was no enhancement on the enhanced scan, and a few showed inhomogeneous ring enhancement or noticeable enhancement. Atypical enterogenous cysts offer a variety of MRI signals due to different protein content, such as hyperintense on T1WI and hypointense on T2WI due to more protein components or intracapsular hemorrhage. This kind of case can only be diagnosed by pathology (26). Epidermoid cysts often occur in the cerebellopontine angle area. Epidermoid cysts tend to surround adjacent blood vessels and cranial nerves. Although, in our case, the MRA showed a posterior fossa mass around the vertebral artery, but there was no restricted diffusion hypointense on the ADC. The typical signs of epidermoid cysts are restricted diffusion on DWI/ADC; the presence or absence of DWI/ADC signal is an important feature in the differential diagnosis of CP and epidermoid cysts (27). Most dermoid cysts showed hyperintense or mixed hyperintense in the T1WI, and few showed hypointense. T2WI mainly showed hyperintense but also showed equal signal or mixed signals. In addition, dermoid cysts showed restricted diffusion in the DWI because they contained sticky, fatty substances and closely arranged cells (28).

To summarize, we report a rare case of ACP involving adults' anterior, middle, and posterior cranial fossa. Its imaging features are not typical. In general, the more significant focus of CP is in the suprasellar cistern. Unlike our case, the tumor in the anterior cranial and posterior cranial fossa is larger and creeping, while those in the suprasellar cistern are smaller. Multilocular cystic ACP involving the anterior, middle, and posterior fossa is rare in adults. This study may be helpful for radiologists in the diagnosis and differential diagnosis of CP by analyzing the imaging features of intracranial cystic tumors.

## Data availability statement

The original contributions presented in the study are included in the article/supplementary material, further inquiries can be directed to the corresponding author.

## Author contributions

BT: manuscript writing. ML: pathological review. XD, HZ, and KZ: manuscript revision. SL: conception and critical review. All authors contributed to the article and approved the submitted version.
